# Effect of Vibration Timing on Mechanical and Durability Properties of Early-Strength Cement-Based Composites for Bridge Wet Joints

**DOI:** 10.3390/ma18204645

**Published:** 2025-10-10

**Authors:** Xiaodong Li, Jianxin Li, Xiang Tian, Yafeng Pang, Bing Fu, Shuangxi Zhou

**Affiliations:** 1 Guangzhou Beierhuan Traffic Technology Co., Ltd., Guangzhou 510700, China; 2 School of Future Transportation, Guangzhou Maritime University, Guangzhou 510700, China; 3 Engineering Research Center for Solid Waste Utilization Towards Green Intelligent Construction, Guangzhou Maritime University, Guangzhou 510700, China; 4 School of Civil Engineering, University of Science and Technology Liaoning, Anshan 114051, China; 5 School of Mechanics and Construction Engineering, Jinan University, Guangzhou 510632, China

**Keywords:** vibration, early-strength, bridge wet joints, mechanical property, durability property

## Abstract

This study explores the influence of vibration timing on the performance of high early-strength cement-based composites used in bridge wet joints. A series of experimental techniques, including SEM, MIP, and RCM tests, were employed to evaluate microstructural evolution, mechanical properties, and durability. The results indicate that vibration applied between the initial and final setting phases has a critical impact, significantly reducing early-age compressive, flexural, and bond strengths. This deterioration is mainly attributed to micro-crack formation and enhanced pore connectivity, as confirmed by SEM and MIP analyses. Moreover, vibration markedly increases the chloride diffusion coefficient, particularly in mixtures with higher water-to-binder ratios, thereby raising long-term durability concerns. These findings underscore the necessity of optimizing mix proportions and strictly controlling vibration timing to ensure both the mechanical performance and service life of high early-strength cement composites in bridge construction. The study provides practical insights for the design and application of durable, resilient bridge wet joints.

## 1. Introduction

The construction of durable and resilient infrastructure has long been recognized as a central challenge in civil engineering. This challenge becomes particularly significant when dealing with structures exposed to aggressive service environments, such as fluctuating temperatures, high humidity, freeze–thaw cycles, chloride-laden deicing salts, and marine conditions [1,2]. Among these structures, bridges occupy a pivotal role due to their constant exposure to both dynamic traffic loads and harsh environmental factors. Any deterioration in bridge performance directly threatens public safety and can lead to costly maintenance and rehabilitation efforts [3]. Within bridge systems, wet joints are used to connect precast segments. They ensure structural continuity and directly influence the durability and service life of the entire bridge. For these joints, achieving high early strength is essential to resist construction loads, allow rapid assembly, and minimize traffic disruption [4,5]. Consequently, the development and application of high early-strength cement-based composites has attracted significant attention in bridge construction. In recent years, a growing body of research has highlighted the superior early load-bearing capacity and durability of high-performance cementitious materials in bridge joints, while also stressing their heightened sensitivity to construction practices [6,7,8]. In particular, under aggressive service conditions, failure to balance rapid construction with adequate material performance may lead to premature damage and undermine long-term service life [9].

Despite the advantages of these composites, their field performance can be strongly influenced by construction practices, particularly vibration. Vibration is an indispensable part of concrete placement and compaction [10]. It helps eliminate entrapped air, improve density, and ensure proper bonding between concrete and reinforcement [11]. However, if vibration is applied during inappropriate stages of the hydration process particularly in the transition from the plastic to the rigid state, which can have detrimental consequences [12]. Instead of improving quality, excessive or poorly timed vibration may cause segregation of constituents, disrupt the developing cementitious matrix, and hinder the formation of a dense microstructure. These disturbances manifest as micro-cracking, increased pore connectivity, and localized weaknesses, which collectively compromise both the short-term mechanical strength and the long-term durability of the material [13]. Recent findings further reveal that poorly controlled vibration may alter the distribution of hydration products, delay or hinder the formation of calcium–silicate–hydrate (C–S–H) gel, and aggravate defects in the interfacial transition zone (ITZ). Such microstructural disturbances can accelerate chloride ingress and exacerbate durability degradation under fatigue loading or cyclic environmental stresses [14,15,16].

The problem becomes more acute when dealing with high early-strength cement-based composites. Unlike conventional concrete, these materials are designed to reach substantial strength within hours or a few days after placement, enabling fast-track construction and early load-bearing [17,18]. Their rapid hydration and accelerated microstructural development are achieved through optimized binder compositions, chemical admixtures, and supplementary cementitious materials. While these features are highly beneficial in practice, they also make the composites more sensitive to external disturbances during the setting and hardening phases [19]. Even minor vibrations in this critical window may lead to significant alterations in the pore structure or hydration products. As a result, the vulnerability of high early-strength composites to vibration is considerably greater than that of conventional concrete, underscoring the importance of precisely controlling vibration timing to preserve the intended mechanical and durability performance [20]. Moreover, compared with ordinary concrete, high early-strength composites often exhibit higher internal temperature rise due to rapid hydration. The resulting thermal gradients can magnify the detrimental effects of vibration on matrix consolidation and crack initiation, making field applications even more complex and difficult to control [21,22]. This sensitivity underlines the need to carefully align vibration practices with the accelerated hydration kinetics of these composites.

Although vibration practices have been extensively studied in the context of ordinary concrete, there remains a noticeable gap in the literature concerning their effects on high early-strength composites, particularly those used in bridge wet joints [23,24]. Most existing studies focus on mix design, hydration kinetics, or durability of these composites, while the interaction between construction methods, such as vibration timing and material performance has received little attention. Given that improper vibration may introduce defects that persist throughout the service life of the joint, the lack of systematic research on this topic represents a significant limitation for both scientific understanding and engineering practice [25,26]. The few available studies have largely been limited to laboratory-scale specimens, with very little evidence addressing real bridge joint conditions. This lack of large-scale and field-oriented research leaves current construction standards without clear guidance on the “safe vibration window” for high early-strength composites [27,28]. With the growing demand for fast-track yet durable bridge construction, optimizing the synergy between material design and construction practices has become a frontier issue in both academia and industry.

To address this critical gap, the present study systematically investigates the impact of vibration timing on the mechanical and durability performance of high early-strength cement-based composites used in bridge wet joints [29,30]. The research pursues three major objectives. First, it evaluates the mechanical properties including compressive, flexural, and bond strength under different vibration timings during the setting and hardening phases. Second, it employs Scanning Electron Microscopy (SEM) and Mercury Intrusion Porosimetry (MIP) to analyze microstructural features, focusing on micro-cracking and pore structure evolution induced by vibration. Third, it assesses durability by measuring resistance to chloride ingress through Rapid Chloride Migration (RCM) tests, which is particularly relevant for bridges exposed to marine or deicing environments [31,32]. By integrating microstructural characterization with macroscopic mechanical and durability evaluations, the study aims to establish a quantifiable vibration timing protocol tailored for high early-strength composites. This approach is expected to provide both theoretical insights and practical construction guidelines, thereby ensuring the dual objectives of rapid construction and long-term durability in bridge wet joints. Based on these investigations, the study aims to identify optimized mix proportions and vibration protocols that enhance both the early-age performance and long-term durability of bridge wet joints. The findings are expected to provide practical guidelines for construction engineers and contribute to the development of more durable, resilient infrastructure.

## 2. Materials and Methods

### 2.1. Materials

In this study, PII 52.5R ordinary Portland cement (OPC) and 72.5 sulfoaluminate cement (SAC) were employed. The Blaine fineness values for OPC and SAC are 389 m^2^/kg and 478 m^2^/kg, respectively. The chemical compositions of both OPC and SAC were determined using X-ray fluorescence (XRF), as detailed in Table 1. The composite admixtures (CAM) consisted of silica fume (SF) and glass microbeads (GMb). The SiO_2_ content in silicon powder exceeds 92%. The D_50_ and D_90_ particle sizes of the SF are 13.38 µm and 30.10 µm, respectively. The glass microbeads were produced by Tianjin Zhicheng New Material Technology Co., Ltd. (Tianjin, China). The particle size distribution of the GMb has D_50_ and D_100_ values of 1.24 µm and 11.94 µm, respectively. The chemical compositions of SF and GMb are shown in Table 1.

The composite additives (CAD) consisted of a water-reducing agent, defoamer, and retarder in precise proportions. The ZJ-PC8800 high-performance polycarboxylate(Quanchi New Materials Co., Ltd, Jinan, China) was used as the water-reducing agent. The ZJ-D130 efficient dry powder defoamer (New Wancheng Technology Co., Ltd, Guangzhou, China), known for its excellent defoaming capability, was employed as the defoamer. Tartaric acid was used as the retarder. The physical properties of the composite additives are shown in Table 2. Additionally, laboratory-grade water and quartz sand with fineness modulus of 2.86 were used for mixing.

### 2.2. Mix Proportion and Sample Preparation

Table 3 delineates the mix proportions of the high early-strength cement composites for bridge wet-joint connection evaluated in this investigation. Four variant formulations were prepared: WJM1, WJM2, WJP1, and WJP2. SAC and CAM constitute 35% of the total binder materials, replacing cement to achieve early strength. Additionally, CAD is included to adjust the properties of the mortars (WJM). The different W/B ratios employed in this study allow for comparative analysis. Mortar samples were prepared for macroscopic tests, while paste samples were prepared for microscopic tests. The mix designs and their specific proportions are detailed in Table 3.

All binder and sand components were thoroughly mixed using a high-shear N50-619 Hobart mixer (Jiangsu Chitong Machinery Canufacturing CO., LTD, Changzhou, Jiangsu, China). Initially, OPC, SAC, CAM, and CAD were blended together at 500 rpm for two minutes to achieve a uniform dry mix. For mortar samples, sand was then added to the dry mixture and mixed at 500 rpm for an additional two minutes. Following this, deionized water was added to the mix. The blending process proceeded at 500 rpm for three minutes, paused for two minutes, and then finished with intense mixing at 2000 rpm for another two minutes to ensure a consistent and homogeneous paste. This final mixture was then cast into molds and securely sealed. For paste samples, the dry mix of OPC, SAC, CAM, and CAD was directly mixed with deionized water, without the addition of sand. The blending process followed the same steps as for the mortar samples: mixing at 500 rpm for three minutes, pausing for two minutes, and then finishing with intense mixing at 2000 rpm for another two minutes. The final paste mixture was then cast into molds and securely sealed. By following these preparation steps, both mortar and paste samples were consistently mixed to ensure uniformity and reproducibility in subsequent testing.

### 2.3. Protocol of Vibration Timing

Current research indicates that the deterioration of concrete due to vibration primarily occurs during the setting and hardening phases. These phases are classified based on the penetration resistance values of the concrete, with the period affecting the concrete’s performance termed the vibration period. According to the national standard “Standard for Performance Test Method of Ordinary Concrete Mix” (GB/T 50080-2016), the setting and hardening stages are defined as follows: the initial setting stage is when the penetration resistance ranges from 0 to 3.5 MPa, the intermediate setting stage ranges from 3.5 MPa to 28 MPa, and the final setting stage is when the penetration resistance exceeds 28 MPa. The intermediate setting stage is the most significantly affected by vibration. As shown in Table 4, the setting and hardening process of the concrete was divided into four protocols based on the vibration period and practical engineering needs: Protocol A, with no vibration; Protocol B, with vibration between the initial to final setting; Protocol C, with vibration 10 min after final setting; and Protocol D, with vibration 3 h after final setting. Concrete was subjected to vibration using an ASR-DC-50C electromagnetic vibration table (Dongguan Lijian Testing Equipment Co., Ltd, Dongguan, China) at a frequency of 15 Hz, an amplitude of 5 mm, and a duration of 10 min.

### 2.4. Experimental Methodology

The mortar samples were used for macroscopic tests, including setting time, workability, strength tests, and chloride tests. The paste samples were used for X-ray diffraction (XRD), mercury intrusion porosimetry (MIP), and scanning electron microscopy (SEM) analyses.

#### 2.4.1. Setting Time and Workability

The setting time of the cement paste was determined using a Vicat apparatus (Cangzhou Dongyi Rongke Experimental Instrument Co., Ltd, Changzhou, China) in accordance with the national standard GB/T 1346-2011: Test Methods for Water Requirement of Normal Consistency, Setting Time, and Soundness of Portland Cement. The initial setting time was defined as the period from the addition of water until the Vicat needle failed to penetrate the bottom plate. Measurements were recorded at 5 min intervals, and at 15 s intervals as the endpoint approached. The final setting time was determined as the point at which the needle no longer left an impression on the specimen surface, with readings taken every 5 min and subsequently every 30 s near the endpoint.

The workability of the mortar was evaluated using the flow table method in accordance with GB/T 17671-2021: Methods of Testing Cement—Determination of Strength. The mortar was placed into a standardized flow table cone mold in two successive layers, each compacted with 25 strokes of a tamper. After compaction, the mold was carefully removed, and the table was dropped 25 times within 15 s. The resulting spread diameter was measured along two perpendicular directions, and the average value was recorded as the flow, which served as an indicator of the mortar’s consistency and flowability.

#### 2.4.2. Compressive, Flexural and Bond Strength Test

The compressive and flexural strength tests were carried out in accordance with the national standard “Test Methods for Strength of Cement Mortar” (GB/T 17671-2021). Prismatic mortar specimens (40 mm × 40 mm × 160 mm) were prepared using a mortar mixer (Zhengzhou Haohai Machinery Equipment Co., Ltd, Zhengzhou, China) and cured to the designated testing age. Prior to testing, surface deposits were removed and the specimens were kept moist. In the flexural test, the side surface formed during molding served as the bearing surface, and loading was applied at a rate of 50 N/s until failure, with the maximum load recorded. The two halves obtained from the flexural test were subsequently used for the compressive strength test, conducted at a loading rate of 2.4 kN/s.

The bond performance between rebar and mortar after vibration was evaluated in accordance with the standard “Test Methods for Physical and Mechanical Properties of Concrete” (GB/T 50081-2019). Test specimens were prepared using HRB400 ribbed steel rebar (Foshan, China) with a diameter of 16 mm and a length of 200 mm, embedded centrally in the mortar. The loading end of the rebar was positioned 100 mm from the mortar surface to facilitate testing.

#### 2.4.3. Microscopic Test

In this study, the microscopic test includes the Rapid Chloride Migration (RCM) test, XRD analysis, MIP analysis and SEM analysis.

The Rapid Chloride Migration (RCM) test was conducted according to the national standard “Test Methods for Long-term Performance and Durability of Ordinary Concrete” (GB/T 50082-2009) using the NTB-DAL meter from Beijing NAILDE (Beijing, China). Cylindrical specimens (100 mm diameter and 50 mm height) were cured for 28 days before testing. The Rapid Chloride Migration (RCM) method was used to determine the chloride ion migration coefficient. A 10% NaCl solution served as the cathode solution, and a 0.3 mol/L NaOH solution served as the anode solution, both stored at 20–25 °C. The moist specimens were subjected to the chloride ion migration test, recording the applied voltage (U), average temperature (T), and test duration (t). After testing, the specimens were split, and the fracture surfaces were sprayed with a 0.1 mol/L AgNO_3_ solution to indicate chloride penetration. The average penetration depth was measured, and the chloride migration coefficient was calculated using NELD-BLO38 software (Beijing, China).

A small portion of the specimen was taken from anhydrous ethanol and dried in a vacuum drying oven. The dried specimen was then ground into a powder using a mortar and pestle, and the powder was evenly spread on a glass slide. The sample was scanned using a SmartLab X-ray diffractometer from Rigaku, Tokyo, Japan, with a scanning range of 5–85° and a scanning rate of 5°/min. The results were processed using HighScore Plus software 5.3 (Beijing, China) to analyze the phase composition.

Mercury intrusion porosimetry (MIP) was used to test the pore structure distribution of concrete mortar. MIP is the most widely used method for determining material pore structures. The basic principle involves forcing mercury, which is non-wetting to most materials, into the specimen’s pores under pressure. The greater the pressure, the more mercury is forced into smaller pores. Conversely, lower pressure fills larger pores. By correlating pore size with applied pressure and measuring the volume of intruded mercury, the pore size distribution is determined.

A small portion of the specimen was taken from anhydrous ethanol and cut into thin slices. These slices were then soaked and cleaned in anhydrous ethanol, followed by drying. The prepared slices were placed on a copper plate with conductive adhesive and coated with gold. The samples were then examined using a Crossbeam 350 focused ion beam scanning electron microscope (FIB-SEM) from Carl Zeiss, Jena, Germany. The SEM analysis was used to observe the hydration products and morphology of the cement mortar.

## 3. Results and Discussion

### 3.1. Workability

**Figure 1:**
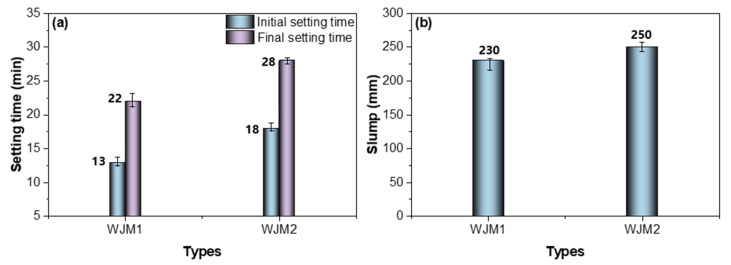
Setting time and slump results of WJM. (**a**) Setting time (**b**) Slump.

To better understand the influence of water dosage and W/B ratio on the fresh properties of the developed mortars, the setting time and slump were first evaluated. These parameters provide essential insights into the workability and early-age behavior of the mixtures, which are critical for ensuring proper placement and compaction in wet-joint applications. Figure 1 presents a comparative analysis of the setting times and slump values for two different types of mortar, WJM1 and WJM2, which differ primarily in their water-to-binder (W/B) ratios and water content, as indicated in Table 3. The initial and final setting times for WJM2 are significantly longer than those for WJM1. Specifically, WJM2 has an initial setting time of 18 min and a final setting time of 28 min, while WJM1 has an initial setting time of 13 min and a final setting time of 22 min. This indicates that the higher water content and W/B ratio in WJM2 result in a longer setting time. Additionally, the slump test results demonstrate that WJM2 exhibits a higher slump value of 250 mm compared to 230 mm for WJM1. This higher slump value suggests that the increased water content in WJM2 enhances its workability and flowability. In summary, the comparative analysis indicates that WJM2, with its higher water content and W/B ratio, not only takes longer to set but also provides enhanced workability compared to WJM1.

### 3.2. Strength Performance

#### 3.2.1. Compressive Strength

**Figure 2:**
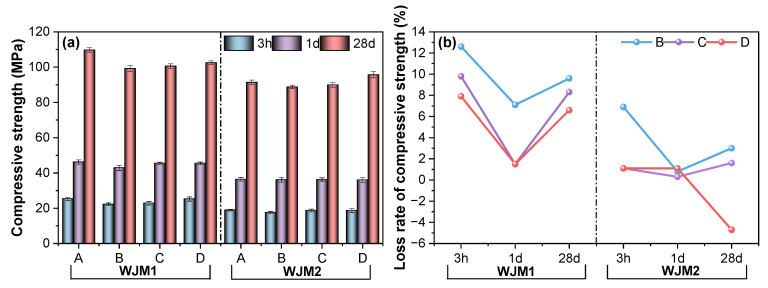
(**a**) Compressive strength of mortar specimens and (**b**) Loss rate of compressive strength of mortar specimens in different protocols of vibration timing.

To evaluate the influence of vibration timing on the mechanical properties, Figure 2 illustrates the compressive strength and loss rate of compressive strength for mortar specimens under different protocols of vibration timing. In Figure 2a, the compressive strength of mortar specimens WJM1 and WJM2 is compared at three different ages: 3 h, 1 day, and 28 days. For both WJM1 and WJM2, the compressive strength increases significantly over time. At 3 h, the compressive strength is relatively low for all specimens. However, by 1 day, there is a notable increase in strength, with further substantial increases observed at 28 days. Among the different vibration protocols (A, B, C, and D), it is evident that WJM2 specimens consistently exhibit higher compressive strengths at each age compared to WJM1 specimens. Protocol A represents no vibration, Protocol B represents vibration between initial to final setting, Protocol C represents vibration 10 min after final setting, and Protocol D represents vibration 3 h after final setting.

In Figure 2b, the loss rate of compressive strength is shown as the percentage of strength relative to Protocol A (no vibration) at the same age. For WJM1, Protocol B shows the highest loss rate at 3 h, which decreases at 1 day and then increases slightly at 28 days. Protocol C shows a more stable trend with minor fluctuations, while Protocol D exhibits the least loss at 28 days. For WJM2, Protocol B shows a gradual decrease in loss rate from 3 h to 28 days. Protocols C and D for WJM2 maintain relatively lower loss rates across all ages, indicating better retention of compressive strength over time.

Overall, the comparative analysis of Figure 2 suggests that WJM2, with its higher water content and W/B ratio, not only achieves higher compressive strength but also demonstrates a lower and more stable loss rate of compressive strength over time. These findings highlight the importance of optimizing vibration timing and mix proportions to enhance the performance and durability of high early-strength cement composites in bridge wet joint connections. Through the loss rate of compressive strength at different vibration timings, it is evident that the initial to final setting period is the most affected by vibration. Therefore, this critical period was chosen to study the impact of vibration on the other strengths of the two mix proportions.

#### 3.2.2. Flexural Strength

**Figure 3:**
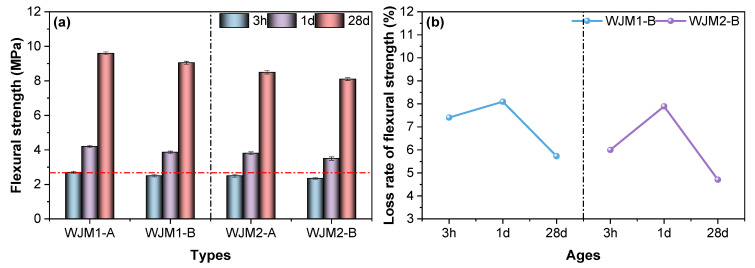
(**a**) Flexural strength of mortar specimens and (**b**) Loss rate of flexural strength of mortar specimens in different protocols of vibration timing.

To further assess the flexural behavior, Figure 3 presents the flexural strength and the loss rate of flexural strength for mortar specimens subjected to different vibration timings, highlighting the significant sensitivity of flexural response to vibration. In Figure 3a, the flexural strength of mortar specimens WJM1 and WJM2 is compared at three different ages: 3 h, 1 day, and 28 days. For both WJM1 and WJM2, the flexural strength increases significantly over time. At 3 h, the flexural strength is relatively low for all specimens. By 1 day, there is a notable increase in strength, with further substantial increases observed at 28 days. Among the different vibration protocols, Protocol A (no vibration) serves as the baseline, showing consistent increases in strength over time. Protocol B (vibration between initial to final setting) demonstrates a considerable reduction in flexural strength compared to Protocol A, indicating that vibration during this period significantly affects the material. Protocol B shows the highest reduction in flexural strength for both WJM1 and WJM2, especially at early ages.

In Figure 3b, the loss rate of flexural strength is presented as the percentage difference in strength relative to Protocol A at the same age. For both WJM1 and WJM2, Protocol B exhibits the highest loss rate, especially at 1 day, indicating that the period between initial to final setting is the most detrimental for vibration effects. The loss rates decrease by 28 days, suggesting some recovery in flexural strength over time. Protocol C (vibration 10 min after final setting) and Protocol D (vibration 3 h after final setting) exhibit lower loss rates, indicating that vibration during these periods has a less significant impact on the flexural strength compared to Protocol B.

This analysis reveals that the period between initial to final setting is the most critical for the impact of vibration on concrete strength. Therefore, the influence of vibration on flexural strength was studied during this most unfavorable period for both mix proportions (WJM1 and WJM2). The results suggest that careful consideration of vibration timing is essential to minimize adverse effects on the mechanical properties of high early-strength cement composites, particularly in bridge wet joint connections.

#### 3.2.3. Bond Strength

**Figure 4:**
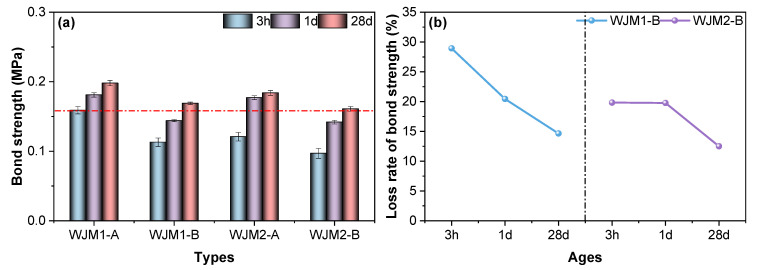
(**a**) Bond strength of mortar specimens and (**b**) Loss rate of bond strength of mortar specimens in different protocols of vibration timing.

In order to examine the interfacial performance between mortar and reinforcement, Figure 4 shows the bond strength and the corresponding loss rate of bond strength under various vibration conditions, providing insight into the effect of vibration timing on mortar–steel adhesion. This figure provides insights into how the timing of vibration impacts the bond strength between the mortar and embedded steel rebar. In Figure 4a, the bond strength of mortar specimens WJM1 and WJM2 is compared at three different ages: 3 h, 1 day, and 28 days. For both WJM1 and WJM2, the bond strength increases over time. At 3 h, the bond strength is relatively low for all specimens. By 1 day, there is a noticeable increase in bond strength, with further significant increases observed at 28 days. Among the different vibration protocols (A and B), Protocol A (no vibration) serves as the baseline and shows a consistent increase in bond strength over time. Protocol B (vibration between initial to final setting) shows a reduction in bond strength compared to Protocol A, indicating that vibration during this critical period negatively affects the bond performance. Notably, the bond strength for WJM2 is generally higher than that for WJM1 across all ages and protocols, which can be attributed to the higher water content and W/B ratio in WJM2.

In Figure 4b, the loss rate of bond strength is presented as the percentage difference in strength relative to Protocol A at the same age. For WJM1, Protocol B exhibits the highest loss rate at 3 h, which gradually decreases at 1 day and 28 days. This trend indicates that the initial impact of vibration on bond strength is significant but lessens over time as the concrete continues to cure and gain strength. For WJM2, Protocol B also shows a high loss rate at 3 h, with a slight decrease at 1 day and a more substantial decrease by 28 days. The loss rates for WJM2 are generally lower than those for WJM1, suggesting better recovery and retention of bond strength over time for WJM2 specimens.

Overall, the comparative analysis of Figure 4 suggests that vibration during the initial to final setting period (Protocol B) has the most detrimental impact on bond strength for both WJM1 and WJM2. However, WJM2, with its higher water content and W/B ratio, demonstrates better performance in terms of higher bond strength and lower loss rates over time. These findings highlight the critical importance of controlling vibration timing to enhance the bond performance and overall durability of high early-strength cement composites in bridge wet joint connections.

### 3.3. Microscopic Performance

#### 3.3.1. Chloride Penetration Resistance

**Figure 5:**
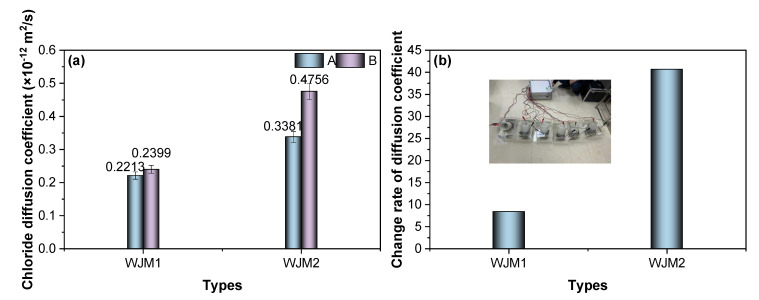
(**a**) Chloride diffusion coefficient of mortar specimens and (**b**) change rate of diffusion coefficient (%) in different protocols of vibration timing.

To investigate the permeability characteristics, Figure 5 depicts the chloride diffusion coefficient and the change rate of diffusion coefficient for mortar specimens subjected to different vibration timings, thereby reflecting the impact of vibration on durability-related transport properties.

In Figure 5a, the chloride diffusion coefficients of mortar specimens WJM1 and WJM2 are compared under two different vibration protocols: A (no vibration) and B (vibration between initial to final setting). For WJM1, the chloride diffusion coefficients are relatively low, with values of 0.2213 × 10^−12^ m^2^/s for Protocol A and 0.2399 × 10^−12^ m^2^/s for Protocol B. This indicates that WJM1 has relatively low permeability, and the effect of vibration on chloride diffusion is minimal. In contrast, WJM2 exhibits significantly higher chloride diffusion coefficients, with values of 0.3381 × 10^−12^ m^2^/s for Protocol A and 0.4756 × 10^−12^ m^2^/s for Protocol B. This suggests that WJM2 has higher permeability, and vibration during the initial to final setting period substantially increases the chloride diffusion coefficient.

In Figure 5b, the change rate of the diffusion coefficient is presented as the percentage increase in chloride diffusion relative to Protocol A. For WJM1, the change rate is relatively low, indicating that the effect of vibration on chloride permeability is minimal. However, for WJM2, the change rate is significantly higher, reflecting a substantial increase in chloride diffusion due to vibration during the critical initial to final setting period. The results show that the permeability of WJM2 is more sensitive to vibration, resulting in higher diffusion rates.

Overall, the comparative analysis of Figure 5 suggests that vibration during the initial to final setting period (Protocol B) has a more pronounced impact on the chloride diffusion coefficient, particularly for WJM2 specimens. The higher permeability observed in WJM2 highlights the need for careful control of vibration timing to mitigate the adverse effects on the durability of high early-strength cement composites, especially in applications such as bridge wet joint connections where chloride penetration can lead to long-term deterioration.

#### 3.3.2. XRD Analysis

**Figure 6:**
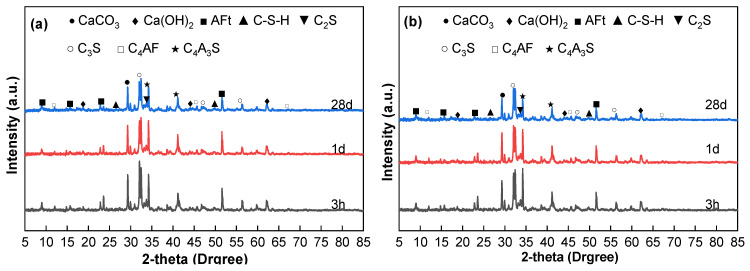

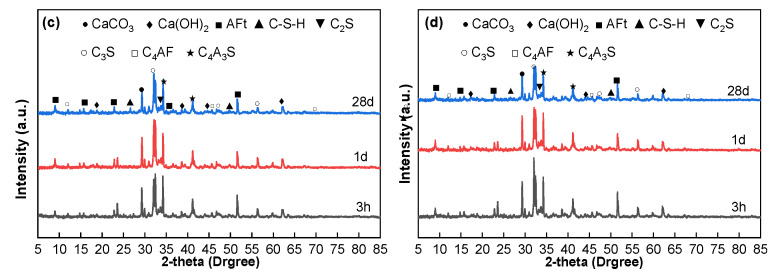
XRD results of (**a**) WJP1-A, (**b**) WJP1-B, (**c**) WJP2-A and (**d**) WJP2-B in different ages.

The analysis of the XRD patterns reveals the evolution of the microstructure during the hydration process. Figure 6 illustrates the XRD results of mortar specimens WJP1-A, WJP1-B, WJP2-A, and WJP2-B at different ages (3 h, 1 day, and 28 days).

According to the XRD patterns, the main mineral components of the concrete before and after vibration include dicalcium silicate (C_2_S), tricalcium silicate (C_3_S), anhydrous calcium aluminate sulfate (C_4_A_3_S), ettringite (AFt), calcium hydroxide (Ca(OH)_2_), tetracalcium aluminoferrite (C_4_AF), calcium carbonate (CaCO_3_), and calcium silicate hydrate (C-S-H). Among these, C_3_S, C_2_S, C_4_A_3_S, and C_4_AF are primarily derived from the unhydrated cement, while AFt, Ca(OH)_2_, CaCO_3_, and C-S-H are the main hydration products.

In Figure 6a, the X-ray diffraction analysis reveals the microstructural evolution of the concrete system during hydration over time. At 3 h, the hydration reaction has just begun, and distinct peaks of C_2_S, C_3_S, and C_4_A_3_S are observed, indicating the active phases at the early stage of cement hydration. AFt, Ca(OH)_2_, and C_4_AF are also present, suggesting that initial hydration products have already formed within the first 3 h. As hydration progresses, the peaks of C_3_S and C_4_A_3_S show significant weakening at 1 day, indicating that these components are actively participating in the hydration reaction, forming C-S-H and CH (Ca(OH)_2_) as hydration products. The formation of AFt, which fills the internal vhoids of the concrete and contributes to early strength, marks the beginning of the hardening process. At 28 days, the diffraction peaks of C_3_S decrease further, while the peaks of C-S-H intensify, indicating that C_3_S has been largely converted to C-S-H, providing major strength to the cement. Additionally, the intensity of the AFt phase decreases slightly, possibly due to the conversion of AFt to AFm (monosulfate) in later stages. Notably, the Ca(OH)_2_ phase shows a significant reduction at 28 days, likely due to the consumption of Ca(OH)_2_ by secondary pozzolanic reactions with added active silica fume (SF) and glass microbeads, which produce additional C-S-H and enhance late-stage strength.

From Figure 6a (WJP1-A, no vibration), the XRD patterns show prominent diffraction peaks of C_2_S, C_4_A_3_S, CaCO_3_, and AFt. With increasing age, the intensities of the diffraction peaks corresponding to C_2_S and C_4_A_3_S decrease, indicating the progression of hydration. The diffraction angles at 2θ = 29.34°, 32.52°, 34.28°, and 51.58° show distinct features, with peaks at 29.34° and 51.58° representing hydration products CaCO_3_ and AFt, respectively, and peaks at 32.52° and 34.28° corresponding to residual unhydrated mineral components C_2_S and C_4_A_3_S.

In Figure 6b (WJP1-B, vibration between initial and final setting), the XRD patterns show that at 3 h, the diffraction peak intensities of CaCO_3_ and AFt are lower than those in the non-vibrated group, which explains the reduced strength observed after vibration. At 1 day, the diffraction peak intensities of CaCO_3_ and AFt are still lower than those in the non-vibrated group, but the reduction is less pronounced, reflecting a lower compressive strength loss rate at 1 day compared to 3 h. At 28 days, the diffraction peaks of various hydration products show no significant changes compared to the non-vibrated group, indicating that vibration has no noticeable impact on the compressive strength at 28 days.

In Figure 6c,d, similar trends are observed for WJP2-A and WJP2-B. The early hydration products and residual unhydrated components follow the same pattern of evolution as seen in WJP1 specimens. The effect of vibration on WJP2 specimens is consistent with the observations in WJP1, where initial vibration impacts early strength development, but the long-term strength at 28 days remains relatively unaffected.

Overall, the XRD analysis indicates that vibration during the initial to final setting period significantly impacts the formation and intensity of early hydration products, affecting early strength. However, the long-term strength at 28 days shows minimal changes, highlighting the resilience of the hydration process over time.

#### 3.3.3. MIP Analysis

**Figure 7:**
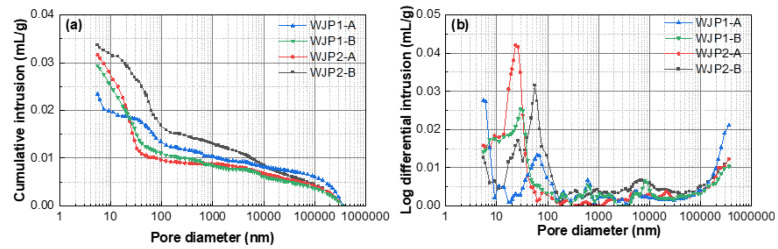
Cumulative intrusion and log differential intrusion of the cement composites at 28 days. (**a**) cumulative intrusion, (**b**) log differential intrusion.

With regard to pore structure evolution, Figure 7 illustrates the cumulative intrusion and log differential intrusion of the cement composites at 28 days, while Table 5 presents the structural parameters of concrete pores under different disturbance conditions. The analysis focuses on the impact of vibration on the pore structure of concrete. The categorization of pore sizes affecting concrete performance divides pores into harmless pores (<20 nm), less harmful pores (20–50 nm), harmful pores (50–200 nm), and more harmful pores (>200 nm). Reducing pores larger than 100 nm is considered beneficial for improving concrete performance. Mercury intrusion porosimetry (MIP) tests provide quantitative information on porosity and pore size distribution, offering a comprehensive and direct reflection of concrete compactness. This study employs MIP to examine the micro-pore structure of concrete specimens at 28 days, both before and after vibration, to understand the impact of vibration on concrete pore structure.

From Table 5, it can be observed that the vibrated groups (WJP1-B and WJP2-B) show an increase in porosity, total pore volume, and most probable pore size compared to the non-vibrated groups (WJP1-A and WJP2-A), while the average pore size decreases. The most probable pore size refers to the diameter of the pores that appear most frequently among all pores, indicating that pores smaller than this size cannot form continuous channels within the cement paste. This suggests that vibration damages the internal pore structure of concrete, increasing pore connectivity and the likelihood of capillary and larger pores. Vibration introduces more air bubbles and pores, causing disordered agglomeration within the concrete, which expands capillary pores. These factors collectively lead to the deterioration of the pore structure in concrete samples after vibration. For WJP2, similar trends are observed: the vibrated group shows an increase in porosity, total pore volume, most probable pore size, and average pore size, with a noticeable decrease in total pore area. This pattern is consistent with WJP1, indicating that vibration damages the internal pore structure of concrete, leading to increased porosity and larger pores.

Figure 7a shows that the cumulative intrusion increases for both optimal (WJP1) and economical (WJP2) groups after vibration, indicating an increase in total pore volume due to vibration. This increase suggests that vibration introduces more pores within the concrete, causing degradation in the micro and interfacial zones, and leading to an increase in capillary pores. The peak value in the middle of Figure 7b represents the most probable pore size. For both WJP1 and WJP2, the most probable pore size shifts to larger diameters, falling within the range of less harmful pores. This shift indicates that vibration negatively impacts the internal pore structure of concrete, increasing the presence of larger pores.

Overall, the MIP analysis reveals that vibration significantly affects the pore structure of concrete, increasing the total porosity, total pore volume, and the size of harmful pores. These changes indicate that vibration can deteriorate the compactness and durability of high early-strength cement composites, particularly in bridge wet joint connections where pore structure integrity is critical.

#### 3.3.4. SEM Analysis

**Figure 8:**
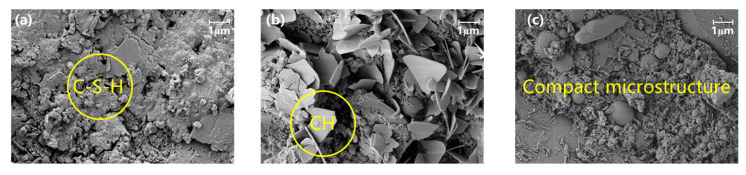
SEM microscopic morphology of the optimal group at different ages: (**a**) 3 h, (**b**) 1 day and (**c**) 28 days.

For microstructural observation, Figure 8 presents the SEM microscopic morphology of the optimal group of concrete at different ages: (a) 3 h, (b) 1 day, and (c) 28 days. The analysis focuses on the hydration products and the development of the microstructure over time, which are crucial for determining the strength and durability of the hardened cement paste.

Hydration products’ bonding degree significantly affects the compactness of the hardened paste, ultimately determining its strength and durability. The experiment utilized SEM analysis to observe the morphology of the concrete hydration products. When cement contacts water, a hydration reaction occurs, releasing a significant amount of heat. Tricalcium silicate (C_3_S), dicalcium silicate (C_2_S), tricalcium aluminate (C_3_A), and tetracalcium aluminoferrite (C_4_AF) dissolve in water, forming an electrolyte solution with Ca^2+^, OH^−^, SO_4_^2−^, Na^+^, and K^+^ ions. The typical hydration products in concrete include fibrous or network-like calcium silicate hydrate (C-S-H) gel, needle-like ettringite (AFt) in the early stages, later appearing as flaky forms, CaCO_3_, Ca(OH)_2_, and unhydrated cementitious materials. The incorporation of microspheres and silica fume does not generate new hydration products but influences the morphology of existing ones.

At 3 h, the SEM image at 10,000× magnification reveals the early stages of the hydration process. A large number of clustered spherical particles and flocculent, loose C-S-H gel are observed on the surface of the hardened paste particles. The C-S-H gel covers the cementitious material particles, forming agglomerated structures that are relatively loose, with visible pores and cracks. The surface of the mortar particles is partially hydrated, but there is still a significant amount of unhydrated cement clinker. This incomplete hydration process contributes to the lower strength of the hardened paste at this early age.

At 1 day, the SEM image at 10,000× magnification shows substantial development in the microstructure. A large number of plate-like Ca(OH)_2_ crystals are observed, indicating the progress of the hydration reaction. The formation of these plate-like crystals suggests that the hydration of C_3_S and C_4_A_3_S is actively occurring, contributing to the development of early strength in the concrete. The structure appears denser than at 3 h, with fewer visible pores and cracks.

At 28 days, the SEM image at 10,000× *g* magnification reveals a well-developed and mature microstructure. The hydration reaction has progressed significantly, resulting in a dense microstructure. The C-S-H gel appears more compact and intertwined, providing the primary source of long-term strength. The microstructure shows fewer pores and a more integrated matrix, indicating that the hydration products have filled the internal voids, enhancing the compactness and durability of the hardened cement paste.

Overall, the SEM analysis indicates that the hydration process leads to significant changes in the microstructure over time. The early stages are characterized by loose and partially hydrated structures, which progressively become denser and more compact as the hydration reaction continues. By 28 days, a well-developed microstructure with minimal porosity and increased strength is observed, demonstrating the importance of hydration products in determining the performance of high early-strength cement composites in bridge wet joint connections.

**Figure 9:**
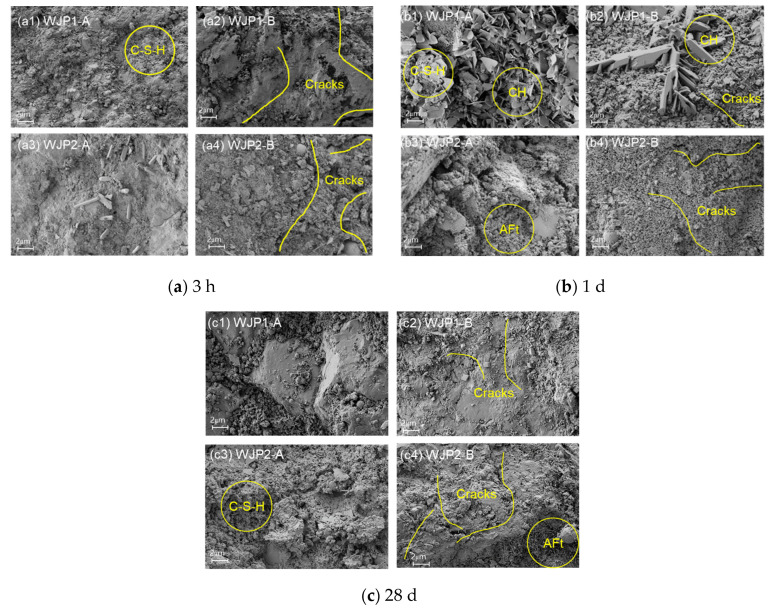
SEM microscopic morphology of WJP1 and WJP2 with vibration or without vibration in different ages: (**a**) 3 h, (**b**) 1 day and (**c**) 28 days.

To provide a comparative perspective, Figure 9 presents the SEM microscopic morphology of WJP1 and WJP2 with and without vibration at different ages: (a) 3 h, (b) 1 day, and (c) 28 days. The analysis focuses on the changes in microstructure due to vibration, highlighting the impact on the strength and durability of the concrete.

At 3 h, the SEM images reveal significant differences between vibrated and non-vibrated samples. In Figure 9(a1,a3) (non-vibrated WJP1 and WJP2), the microstructure shows the initial formation of hydration products such as C-S-H gel and needle-like ettringite (AFt). These products cover the surfaces of cement particles, indicating the early stages of hydration. However, in Figure 9(a2,a4) (vibrated WJP1 and WJP2), numerous cracks and pores are visible in the hardened paste. The presence of these micro-cracks and pores results from the disruptive effect of vibration, which leads to a decrease in early-age strength. The vibrated samples show more pronounced cracks and porosity, which compromises the initial structural integrity of the concrete.

At 1 day, the SEM images show further development in the hydration process. In Figure 9(b1,b3) (non-vibrated WJP1 and WJP2), a substantial amount of granular C-S-H gel, some needle-like AFt, and plate-like Ca(OH)_2_ are observed. These hydration products fill the pores between cement particles, leading to a denser microstructure and increased strength. In contrast, Figure 9(b2,b4) (vibrated WJP1 and WJP2) still exhibit micro-cracks and pores, although the structure is somewhat denser than at 3 h. The cracks and pores are less pronounced than at 3 h but still present, indicating that vibration continues to negatively impact the microstructure, resulting in lower strength compared to non-vibrated samples.

At 28 days, the SEM images demonstrate a well-developed and mature microstructure. In Figure 9(c1,c3) (non-vibrated WJP1 and WJP2), the microstructure is dense and compact, with a significant presence of C-S-H gel and well-formed hydration products. The hydration process has progressed to a stage where the internal voids are mostly filled, providing high strength and durability. In Figure 9(c2,c4) (vibrated WJP1 and WJP2), although the overall structure is denser compared to earlier ages, micro-cracks are still visible. The presence of these fine cracks indicates that vibration causes lasting damage, but its impact is less severe at this mature stage compared to earlier ages.

The SEM analysis reveals that vibration significantly affects the microstructure of concrete at different ages. At 3 h, vibration introduces numerous cracks and pores, weakening the early-age strength. By 1 day, although the microstructure becomes denser, vibrated samples still show micro-cracks and pores, leading to reduced strength compared to non-vibrated samples. At 28 days, the impact of vibration diminishes, with both vibrated and non-vibrated samples exhibiting dense microstructures. However, fine cracks remain in the vibrated samples, indicating some lasting damage. Overall, the analysis highlights the importance of controlling vibration timing to minimize adverse effects on the microstructure and ensure the durability and strength of high early-strength cement composites in bridge wet joint connections.

## 4. Conclusions

This study systematically investigated the influence of vibration timing on the mechanical and durability performance of high early-strength cement-based composites used in bridge wet joints. The main conclusions can be drawn as follows:

(1) Vibration occurring between the initial and final setting periods has a pronounced adverse effect on the early-age mechanical properties, including compressive, flexural, and bond strengths. This finding emphasizes the necessity of minimizing or controlling vibration during this critical hydration stage to ensure structural integrity.
(2) Microstructural characterization (SEM and MIP) demonstrated that vibration induces microcracks and increases pore connectivity in the cement matrix, leading to reduced compactness and long-term durability. Although partial recovery occurs with further hydration, the persistence of fine cracks and elevated porosity highlights the lasting influence of early vibration.
(3) Chloride penetration tests confirmed that vibration significantly elevates the chloride diffusion coefficient, particularly in mixtures with higher water-to-binder ratios (WJM2). This increased permeability accelerates the risk of chloride ingress and reinforcement corrosion, underscoring the importance of vibration control in durability design.
(4) Comparative evaluation of different mix proportions under various vibration protocols revealed that mixtures with higher water content (WJM2) exhibit relatively better resistance to strength loss. Nevertheless, the detrimental effects of vibration remain evident, suggesting that optimization of both mix design and vibration timing is essential for achieving durable and reliable bridge wet joint connections.

Overall, the findings provide new insights into the interaction between vibration timing and material performance, offering practical guidance for the design, construction, and durability management of high early-strength cement composites in bridge engineering applications.

## Figures and Tables

**Table 1:** Chemical compositions of raw materials.

Chemical	CaO	SiO_2_	Fe_2_O_3_	MgO	Al_2_O_3_	K_2_O	Na_2_O	SO_3_	TiO_2_	MnO	Others
OPC	62.52	19.66	3.37	0.85	4.29	0.62	0.08	2.61	0.24	0.17	5.59
SAC	43.40	8.50	1.90	1.80	33.60	—	—	8.20	1.50		1.1
SF	—	93	—	—	—	—	—	—	—		7
GMb	8	55	5	—	20	—	—	3	—		9

**Table 2:** Physical properties of composite additives.

Agents	Appearance	Bulk Density (g/L)	Fineness (Passing 0.3 mm Sieve) (%)	Residual Moisture (%)	Water Reduction Rate (%)	Dry Loss (%)	Residual on 40 Mesh Sieve (%)	Ash Content (%)
Defoamer	White powder	500–700	≥90	≤3	≥25	—	—	—
Water-Reducing Agent	White powder	500–700	≥90	≤3	≥25	—	—	—
Retarder	—	—	—	—	—	1.26	0.39	3.12

**Table 3:** Mix proportions of high early-strength cement composites for Bridge wet-joint connection (kg/m^3^).

Type	Cement	SAC	CAM	Water	Sand	CAD	W/B
WJM1	365	91	104	95.2	560	9.1	0.17
WJM2	365	91	104	106.4	560	9.1	0.19
WJP1	365	91	104	95.2	—	9.1	0.17
WJP2	365	91	104	106.4	—	9.1	0.19

Note: The CAM consists of GMb at 80 kg/m^3^ and SF at 24 kg/m^3^. The CAD consists of a water-reducing agent at 6.7 kg/m^3^, a defoamer at 1.4 kg/m^3^, and a retarder at 1.2 kg/m^3^. “M” in WJM stands for mortar, and “P” in WJP stands for paste.

**Table 4:** Protocol of vibration timing.

Type	No Vibration	Vibration for 10 min		
		Between Initial Setting to Final Setting	10 min After Final Setting	3 h After Final Setting
WJM1, WJM2, WJP1 or WJP2	A	—	—	—
	—	B	—	—
	—	—	C	—
	—	—	—	D

Note: “N” stands for the types WJM1, WJM2, WJP1 or WJP2, while “A,” “B,” “C,” and “D” represent different vibration timings.

**Table 5:** Structural parameters of concrete holes for disturbance tests.

Types	Porosity (%)	Total Porosity Volume (mL/g)	Total Porosity Area (nm)	Most Probable Pore Size (nm)	Average Pore Size (nm)
WJP1-A	5.5638	0.0234	3.074	5.48	30.50
WJP1-B	6.8259	0.0294	4.997	55.78	23.51
WJP2-A	7.2592	0.0316	5.782	23.41	21.88
WJP2-B	7.7014	0.0337	3.013	29.03	44.78

## Data Availability

The original contributions presented in this study are included in the article. Further inquiries can be directed to the corresponding authors.
